# Awareness, Actions, and Predictors of Actions on Adverse Drug Reaction Reporting among Patients Attending a Referral Hospital in Southern Highland Tanzania

**DOI:** 10.1155/2023/7761649

**Published:** 2023-05-09

**Authors:** Nathanael Sirili, Manase Kilonzi, Dorkasi L. Mwakawanga, Juma A. Mohamedi, Joseph Matobo Thobias, Aurelia Clement, Davance Mwasomola, Stella E. Mushy

**Affiliations:** ^1^School of Public Health and Social Sciences, Muhimbili University of Health and Allied Sciences, P.O. Box 65015, Dar es Salaam, Tanzania; ^2^School of Pharmacy, Muhimbili University of Health and Allied Sciences, P.O. Box 65013, Dar es Salaam, Tanzania; ^3^School of Nursing, Muhimbili University of Health and Allied Sciences, P.O. Box 65004, Dar es Salaam, Tanzania; ^4^School of Medicine, Muhimbili University of Health and Allied Sciences, P.O. Box 65001, Dar es Salaam, Tanzania; ^5^Pharmacy Department, Mbeya Zonal Referral Hospital, P.O. Box 419, Mbeya, Tanzania

## Abstract

**Purpose:**

This study assessed the awareness, actions, and predictors of actions on adverse drug reaction reporting among patients attending a referral hospital in southern highland Tanzania.

**Methods:**

A hospital-based cross-sectional study was conducted from January to August 2022 at Mbeya Zonal Referral Hospital (MZRH) in Mbeya, Tanzania. A total of 792 adult patients with chronic conditions attending outpatient clinics at MZRH were recruited consecutively. A semistructured questionnaire was used to collect demographic characteristics, ADR awareness, and actions when encountering ADR. Data were analyzed using the statistical package for social sciences (SPSS) version 23 and results are summarized using frequency and percentages. Binary logistic regression was used to assess the predictors associated with reporting ADR among patients. *P* value ≤0.05 was considered statistically significant.

**Results:**

Out of 792, 397 (50.1%) were males and 383 (48.6%) had a primary education level. Only 171 (21.6%) participants previously experienced ADR, and 111 (14.1%) were aware that ADR is an unexpected harm that occurs after medication use. The majority 597 (70.3%) of the participants said will report ADR to healthcare providers, 706 (88.9%) prefer reporting ADR to healthcare providers, and 558 (69.1%) said patients are not aware of the importance of reporting ADR. Patients aged below 65 years of age, unemployed ((AOR (95% CI) = 0.4 (0.18–0.87), self-employed ((AOR (95% CI) = 0.5 (0.32–0.83)), and those who ever encountered ADR ((AOR (95% CI) = 0.1 (0.05–0.11)) were more likely to report the ADR to HCPs compared to the rest.

**Conclusions:**

The majority of patients are not aware of what is ADR and the importance of ADR reporting. Most of the patients prefer to report ADR to healthcare providers. We recommend an awareness campaign to raise awareness of the patients on ADR and other methods of ADR reporting.

## 1. Introduction

Increases in medication access are associated with incidences of adverse drug reactions (ADRs) to consumers. World Health Organization (WHO) defines ADR as a response to a drug that is noxious and unintended which occurs at a standard dose used for prophylaxis, diagnosis, treatment of a disease, or modification of a physiological function [1]. In European Union, ADR causes about 200,000 deaths and cost around Euro 79 billion annually [2] and in England, ADRs contribute to 16.5% of all hospital admission and a projected annual cost of 2.21 billion [3]. ADR generally results in additional treatment costs for patients and the healthcare system, as well as prolonged hospital stays, rehospitalizations, morbidity, and mortality [4, 5]. ADR monitoring is a component of pharmacovigilance [6]. Pharmacovigilance (PV) is the science related to detecting, assessing, understanding, and preventing ADR and other drug-related problems [7].

Monitoring of safety following the registration of medicine is very important to identify unknown and unusual ADRs. No specific populations, such as children, pregnant women, or the elderly, are included in clinical trials done to register a drug for human use [8, 9]. In addition, the number of participants in clinical studies is insufficient to draw definitive conclusions about medication safety [8, 9]. Nevertheless, off-label uses of medication increase the risk of ADR. Monitoring ADRs facilitates the withdrawal of potentially unsafe medicines from the market [10]. Despite its necessity, underreporting of ADRs is a major public global issue [11, 12].

Underreporting delays the detection of ADRs which causes patient suffering and increases morbidity and mortality rates [13]. WHO recommends a minimum of 200 reported cases per 1,000,000 population for effective monitoring of a single medication ADR [8]. To maximize reporting, the WHO suggests spontaneous reporting of ADRs (SR-ADRs) from healthcare providers (HCPs) and consumers of medications [7, 13]. Previously, SR-ADRs involved only HCPs but literature shows that HCPs report only major and unexpected adverse events. In addition, the literature indicates that HCPs both overestimate and underestimate the importance and relevance of ADR to patients [14]. Therefore, involving consumers maximizes reporting of major, minor, and very rare ADRs that may trigger patients to stop taking medication on time [14].

In developed countries, consumer/patient reporting systems started in the 1960s, however, the practice is new in low-middle-income countries [15]. In many countries, lack of resources and financial constraints has been associated with the poor promotion of consumer reporting systems [6, 12, 16]. Reported barriers to ADR reporting include lack of interest, reluctance, complacency, ignorance, and lack of incentives [12]. In addition, insufficient awareness and knowledge of how to report and the absence of ADR reporting forms have been linked to a low reporting rate [5].

In Tanzania, the Ministry of Health, through the Tanzania Medicine and Medical Devices Authority (TMDA), introduced an online reporting system in October 2016. However, the paper-based reporting system has been in place since 1987. The system allows patients, consumers, and HCPs to access and resubmit reporting forms online (https://www.tmda.go.tz/pages/paper-based-system-for-submission-of-adverse-drug-reaction). To assist those without Internet access, in 2018 TMDA introduced a dial number ^*∗*^152^*∗*^00# through which consumers and HCPs can access reporting forms or text messages. To create awareness of the available ADR reporting platform, TMDA has been actively providing education to HCPs and the public through various platforms, including seminars and radio/television advertisements. To strengthen the reporting system, TMDA appointed a PV focal person in each of the five zones of Tanzania. The PV focal person is responsible for coordinating all the PV-related activities, such as training the HCPs, raising awareness among the public, and distributing of yellow forms in their respective zones. Despite all the efforts, ADRs continue to be underreported in Tanzania. Therefore, this study assessed the awareness, actions, and predictors of actions on adverse drug reaction reporting among patients attending a referral hospital in southern highland Tanzania.

## 2. Methods

### 2.1. Study Design and Study Setting

A hospital-based cross-section study was conducted between January and August 2022 at Mbeya Zonal Referral Hospital (MZRH) in southern highlands, Tanzania. The study was designed to assess the awareness of patients on ADR reporting. MZRH serves patients from 6 regions of Tanzania (Songwe, Mbeya, Iringa, Njombe, Rukwa, and Katavi). Also, most PV activities in the southern highlands zone are organized at MZRH and the PV focal person is an MZRH employee. MZRH serves about 800–1000 outpatients per day.

### 2.2. Study Population

Patients with chronic diseases on medication for at least 3 months who attended the clinic at MZRH with age ≥18 years were asked to participate in this survey. Those who agreed and provided written informed consent were recruited and invited to respond to the survey questions.

### 2.3. Sample Size, Sample Size Calculation, and Sampling Technique

The study adopted a multistage cluster sampling strategy where in the first stage the southern highland zone was randomly selected among the six geopolitical zones in Tanzania. In the second stage, a purposive sampling strategy was used to include MZRH as the only zone referral hospital catering to the zone. In the third stage, we randomly include 5 clinics (internal medicine, care, and treatment clinic (CTC), urology, and orthopedic) from over 12 outpatient clinics, available at MZRH. In the fourth stage, we systematically sampled the patients where the nth (144,000/798) patient was included.

The sample size was calculated using a Yamane formula for finite population *n* = *N*/(1 + *Ne*^2^) whereby *N* = study population and *e* stand for margin of error [17]. At MZRH approximately 900 patients are attending outpatient clinics per day and the clinics run for five days a week. The study duration was eight (8) months, and the expected number of patients (*N*) was 144,000. Assuming a margin of error of 5% and a design effect of 2 we obtained a minimum sample size of 798. Study participants were recruited consecutively in each respective clinic. The study recruited adults above 18 years of age with a diagnosis of chronic diseases on medication attending the selected clinics at MZRH.

### 2.4. Data Collection Procedure

A questionnaire with structured questions was adapted from a study conducted in Nigeria to capture patients' demographic characteristics and awareness of ADR reporting (2019) [5]. The questionnaire contained three sections that collected patients' demographic information (age, gender, marital status, level of education, and occupation), awareness of ADR, pharmacovigilance activities, and reporting of ADR practices. From Nigeria questionnaires few edits were done to make the questions understandable to our participants and researcher assistants. We removed issues of ethnicity from demographic and the questions which requested long-answer were made to be multiple choices (https://doi.org/10.1186/s12913-019-4775-9). The questionnaire used in this study is attached as supplementary material for more references.

Two research assistants (RAs) who were registered pharmacists working at MZRH were recruited and trained for data collection. Training of the RAs was done by a principal investigator who holds a master of science in Pharmacology and Therapeutics in collaboration with zonal pharmacovigilance focal person. They understood the study objectives, the questionnaire, the research process, and the research ethics including the informed consent for participation. The patients responded to survey questions through one-to-one interviews with the RAs at the respective clinic waiting for areas. Before the data collection process commenced, RAs subjected the questionnaire to 10 patients to see the readability of the questions. The challenges were communicated back to investigators and corrections were made up accordingly.

### 2.5. Data Analysis

Statistical package for social sciences version 23 was used to analyze data. The findings were summarized by using frequency and percentages. Age was summarized using mean (± standard deviation). The chi-square test was used to check the determinants of reporting ADR to HCPs among participants. Variables that showed to be significant in the chi-square test were entered in the logistics regression model in blocks. We started with demographic characteristics, then heard of pharmacovigilance, and finally history of encountering ADR. A *P* value of less than 0.05 was considered significant.

## 3. Results

### 3.1. Demographic Characteristics of the Study Participants

The mean (± standard deviation) age of the study participants was 46.4 (±15.2), 397 (50.1%) males, and the majority 472 (64.7%) were married. Participants aged 56–65 years were 168 (21.7%), 383 (48.6%) had a primary education level and 406 (51.7%) were self-employed (Table 1).

### 3.2. Participants' Awareness of ADR, Serious ADR, and ADR Reporting

Only 694 (89.3%) of participants had heard of the ADR reporting form and 171 (21.6%) previously experienced ADR. Only 111 (14.1%) participants were aware that ADR is an unexpected harm that occurs after medication use. More than half 416 (52.7%) felt that serious ADR requires treatment and the majority 597 (70.3%) said they would notify HCPs if they encountered ADR. The majority 706 (88.9%) preferred to report ADR to HCPs as one of the reporting methods, while 558 (69.1%) did not report the ADR because they were unaware of its significance (Table 2).

### 3.3. Actions to be Taken by Participants When Encountering ADR

The majority 514 (68.2%) of the participants said will report the ADR to HCPs, while 240 (31.8%) will take other actions, such as discontinuing the drug(s), doing nothing because the reaction was tolerable, doing nothing because the reaction resolved on its own, using another drug to treat symptoms of the reaction, switching to herbal/traditional medicines, and Switching to a different drug. Most participants received information regarding pharmacovigilance (63.5%) and the yellow form (57.8%) from the HCPs, as shown in (Table 3).

### 3.4. Predictors of the Action to be Taken When Encountering ADR among the Study Participants

Following multivariate binary logistic regression those patients aged below 65 years of age, unemployed ((AOR (95% CI) = 0.4 (0.18–0.87), self-employed ((AOR (95% CI) = 0.5 (0.32–0.83)), and those who ever encountered ADR ((AOR (95% CI) = 0.1 (0.05–0.11)) were more likely to report the ADR to HCPs compared to the rest. However, those who heard about PV ((AOR (95% CI) = 1.8 (1.16–2.78)) were 1.8 likely to opt for the incorrect action and the difference was significant (Table 4).

## 4. Discussion

This study aimed to assess the awareness of the patients regarding ADR reporting in Tanzania. In this study, 64.4% of patients had heard of PV, while only 10.7% had heard of the reporting form, only 14.1% correctly defined ADR, and 21.6% had previously experienced ADR. The majority of the patients received information about PV and reporting forms from HCPs, and reporting ADRs to HCPs was the most action patients took when encountering ADR. The majority of patients do not know the importance of reporting ADRs. Age, occupational status, and prior ADR encounters determined the action to be taken when encountering ADR.

Regarding awareness of PV, our findings differ from those reported in Nigeria and Malaysia, where 8.6% and 8% of patients, respectively, were aware of PV and ADR reporting systems [5, 18]. The disparity in awareness levels observed may be due to the varying levels of efforts undertaken by the respective authorities in each country to educate consumers and the general public about PV. The on-the-job training for HCPs and the appointment of a zonal PV focal person by the responsible authorities in Tanzania contributed to the spread and rise of HCPs awareness, which indirectly influences the awareness of patients/consumers. Similarly, to what was reported in Nigeria [5], the majority of patients claimed that they got information about PV from HCPs, followed by radio and television media.

This study indicates that patients prefer to report ADRs to HCPs. The findings are similar to what was reported in Nigeria, Malaysia, and Thailand, where the majority of patients report ADRs through HCPs [5, 18, 19]. The reporting of ADRs to HCPs should be encouraged whenever possible since the affected individual will receive immediate attention. However, studies show that HCPs have a tendency to underrate or overrate some patients' reports [14]. Therefore, more effort should be devoted to educating patients and the general public about the various methods of reporting ADRs and the available tools and platforms for reporting ADRs directly without the involvement of HCPs. The literature emphasizes that for a patient reporting system to be successful, patients must be adequately educated on the existing pharmacovigilance system and reporting mechanisms for ADRs [20].

Similarly, this study also found that the majority of patients are not aware of the importance of reporting ADRs. In countries where the patient reporting system is well established and effective, it noted that raising awareness among patients/consumers about the significance of reporting and encouraging their participation in medication safety are crucial to the success of the program [21]. In addition, a lack of knowledge, difficulty with reporting procedures, and a lack of feedback have been cited as bottlenecks in consumer reporting [6, 16]. The findings necessitate the use of various available platforms, including social media, television, and radio to educate the public and consumers regarding PV and the importance of reporting ADRs [5, 14, 18]. However, social media use should be approached with caution because, according to some reports, consumers find it difficult to differentiate between correct and incorrect information.

Moreover, in this study, the majority of patients failed to correctly define ADR. Most patients are confused ADR, side-effects, and adverse events. Similar results were observed in a study conducted in Nigeria, in which only 39.7% correctly defined the ADR [5]. Low knowledge of what an ADR is may be the reason why studies indicate that ADR reports directly from patients/consumers are of poor quality [20]. Low knowledge of ADR may also explain why most patients/consumers prefer to report ADR to HCPs over other reporting channels. Therefore, to increase the number of ADR reports from patients/consumers, the community should be educated more. The latter will improve the quality of ADR reports submitted through the various available platforms.

## 5. Limitation and Mitigation

The study was conducted in one health facility and the patients were asked to remember if they have been suffering from ADRs which may limit the generalizability of our findings and impose recall and social desirability bias, respectively. However, the selected health facility is a zonal referral hospital that serves patients from about six regions of Tanzania. The patients who participated in this study as the ones with chronic diseases, attending clinics and on medications for at least 3 months to minimize recall bias. Regarding social desirability biases, research assistants (RAs) were trained on the appropriate way to ask questions while keeping them neutral, unbiased, and nonthreatening to avoid making participants feel threatened or embarrassed when answering questions. RAs also ensured confidentiality by observing anonymity, as well as establishing a good rapport with patients to put participants more at ease.

Additionally, the study did not explore the reasons behind the lack of awareness among patients and did not investigate the quality of patients' filled ADR report forms, potential barriers, and facilitators to ADR reporting among patients. Therefore, further qualitative research may be needed to identify the factors that influence ADR reporting among patients and a document review approach to assessing the quality of patients' reported ADR.

## 6. Conclusion

The majority of patients are not aware of what ADR is and the importance of ADR reporting. Most patients prefer to report ADR to healthcare providers. Age, occupation, and experience with ADR determine the action to be taken when a patient encounters ADR. We recommend an awareness campaign to raise awareness of the patients on ADR and other methods of ADR reporting.

## Figures and Tables

**Table 1:** Demographic characteristics of the study participants.

Variables			*n* (%)
*Sex (n* *=* *792)*			
Male			397 (50.1)
Female			395 (49.9)
Mean age (± standard deviation)	46.4 (±15.2)		

*Age (773)* ^*∗*^			
≤25			64 (8.3)
26–35			162 (21)
36–45			160 (20.7)
46–55			132 (17.1)
56–65			168 (21.7)
>65			87 (11.2)

*Marital status (n* *=* *729)*^*∗*^			
Married			472 (64.7)
Single			257 (35.3)

*Occupation (n* *=* *786)*^*∗*^			
Unemployed			297 (37.8)
Self-employed			406 (51.7)
Civil servant			83 (10.5)

*Education level (788)* ^*∗*^			
Informal			165 (20.9)
Primary			383 (48.6)
Secondary			160 (20.3)
College + university			80 (10.2)

^*∗*^Missing information (age = 19, marital status = 63, occupation = 6, and education level = 4).

**Table 2:** Showing participants' awareness of ADR, serious ADR, and ADR reporting.

Questions	Response *n* (%)
*Ever heard of pharmacovigilance? (n* *=* *787)*^*∗*^	
Yes	507 (64.4)
No	280 (35.6)

*Ever heard of ADR reporting form? (777)* ^*∗*^	
Yes	83 (10.7)
No	694 (89.3)

*Ever encounter ADR? (792)*	
Yes	171 (21.6)
No	621 (78.4)

*Meaning of adverse drug events (n* *=* *790)*^*∗*^	
Any harm related to the use of a drug (adverse event)	298 (37.7)
Expected harm after using a drug (side-effect)	190 (24.1)
Unexpected harm after using a drug (adverse drug reaction)	111 (14.1)
I don't know	191 (24.2)

*Meaning of serious adverse drug reaction (n* *=* *807)*^*∗*^	
A reaction that may lead to hospitalization	118 (14.6)
A reaction that is life-threatening	161 (20.0)
A reaction that requires another drug treatment	416 (52.7)
A reaction that resolves on its own	112 (13.9)

*What action did you or will you take after encountering ADR* ^*1*^^*∗*^	
Inform a healthcare professional	597 (70.3)
Stop the drugs	123 (14.5)
Nothing because the reaction was tolerable	28 (3.3)
Nothing because the reaction resolved on its own	17 (2.0)
Use another drug to treat symptoms of the reaction	47 (5.0)
Switch to herbal/traditional medicines	2 (0.2)
Switch to another drug	40 (4.7)

*Preferred methods of ADR reporting* ^*1*^^*∗*^	
Reporting directly to healthcare professional	706 (88.9)
A phone call or text message	57 (7.2)
Online application designed for adverse drug reaction reporting	10 (1.3)
Filling out a reporting form	21 (2.6)

*Why patients do not report encountering ADRs* ^*1*^^*∗*^	
Do not know the importance of reporting adverse drug reactions	558 (69.1)
The adverse reaction may not be very serious	43 (5.3)
Do not know how to report such reactions	131 (16.2)
Not sure if an adverse reaction is related to the medications used	67 (8.3)
Adverse effects/reactions resolved on their own	9 (1.1)

^1^One individual may answer more than one response. ^*∗*^Missing information.

**Table 3:** Shows source of patients' information regarding pharmacovigilance and yellow form.

Source of information	Pharmacovigilance *n* (%)	Yellow form *n* (%)
Healthcare providers	324 (63.5)	48 (57.8)
Radio	115 (22.5)	4 (4.8)
Television	26 (5.1)	9 (10.8)
Social media	29 (5.7)	7 (8.4)
Newspapers	5 (1)	3 (3.6)
Friends/relatives	10 (2)	11 (13.3)
College/university	1 (0.2)	1 (1.2)

**Table 4:** Binary logistic regression shows predictors of the correct action to be taken when encountering ADR among the study participants.

Variables	COR (95% CI)	*P*value	AOR (95% CI)	*P* value
*Sex*				
Male	0.9 (0.67–1.23)	0.52		
Female	1			

*Age (years)*				
≤25	0.5 (0.27–0.98)	0.04	0.3 (0.12–0.75)	0.01
26–35	0.6 (0.34–1.04)	0.07	0.6 (0.34–1.14)	0.19
36–45	0.8 (0.49–1.21)	0.26	0.6 (0.34–1.12)	0.58
46–55	0.6 (0.36–0.92)	0.02	0.5 (0.27–0.87)	0.02
56–65	0.5 (0.31–0.84)	0.01	0.4 (0.22–0.75)	<0.01
>65	1		1	

*Marital status*				
Married	0.8 (0.61–1.17)	0.31		
Not married	1			

*Occupation*				
Unemployed	0.7 (0.38–1.14)	0.14	0.4 (0.18–0.87)	0.02
Self-employed	0.7 (0.50–0.96)	0.03	0.5 (0.32–0.83)	0.01
Employed	1		1	

*Education status*				
Informal	1.6 (0.85–2.84)	0.15	1.6 (0.74–3.42)	0.24
Primary	1.3 (0.80–2.19)	0.27	0.9 (0.43–1.79)	0.72
Secondary	1.7 (1.10–2.62)	0.02	1.6 (0.90–2.81)	0.11
College/university	1		1	

*Heard of PV*				
Yes	1.4 (0.99–1.89)	0.05	1.8 (1.16–2.78)	0.01
No	1		1	

*Heard of the yellow form*				
Yes	0.8 (0.49–1.27)	0.32		
No	1			

*Ever suffer ADR*				
Yes	0.1 (0.08–0.17)	<0.01	0.1 (0.05–0.11)	<0.01
No	1			

## Data Availability

The datasets used and/or analyzed during the current study are available from the corresponding author upon reasonable request.

## Additional Points

(1) About 89% had heard of ADR reporting forms, among studied participants 14.1% were aware that ADR is an unexpected harm that occurs after medication use, and the majority (88.9%) preferred to report ADR to healthcare providers (HCPs) as one of the reporting systems. (2) The majority (68.2%) likes to report ADR to HCPs, and being unaware of its ADR reporting significance is the reason why the majority are not reporting ADR. (3) Therefore, the majority of patients are not aware of ADR and the importance of reporting ADR. We recommend campaigns to raise awareness of the patients on ADR reporting.

## Supplementary Materials

Supplementary Materials Questionnaire used to collect information from the study participants.
