# Supplementary material for: Awareness, Actions, and Predictors of Actions on Adverse Drug Reaction Reporting among Patients Attending a Referral Hospital in Southern Highland Tanzania
Source: Adv Pharmacol Pharm Sci. 2023 May 9;2023:7761649. doi: 10.1155/2023/7761649 (PMC10188260; doi:10.1155/2023/7761649)
Supplement: Supplementary Materials — Questionnaire used to collect information from the study participants. [file 7761649.f1.doc]

**QUESTIONNAIRE ON KNOWLEDGE, AWARENESS, ATTITUDE AND PRACTICES OF ADVERSE DRUG REACTION REPORTING AMONG PATIENTS**

Dear Sir/Ma,

This questionnaire seeks to assess knowledge, awareness, and practices of adverse drug reactions (ADRs) among patients attending Mbeya Zonal and Regional Referral Hospitals in Mbeya Region, Tanzania. Kindly help to respond to the questionnaire with utmost sincerity. Your response will be anonymous and confidentiality is guaranteed.

Thank you.

**SECTION A- DEMOGRAPHIC INFORMATION**

1. Sex: Female ( ) Male ( )
2. Age:
3. Marital status: Single ( ) Married( ) Other( )
4. Occupation: Civil servant ( ) Self-employed ( ) Unemployed ( )
5. Level of Education: Primary ( ) Secondary ( ) College/University ( ) Informal ( )

**SECTION B: ASSESSMENT OF PATIENT’S KNOWLEDGE/AWARENESS OF PHARMACOVIGILANCE/ADVERSE DRUG REACTIONS**

**INSTRUCTION**: Please tick or circle the appropriate responses as it applies to you. Please note **that you can select more than one option**

1. Which of the following best describes your understanding of adverse drug reactions?
2. Any effect from a medication one is using
3. Unexpected reaction after taking a drug
4. Expected reaction after taking a medicine
5. I don’t know
6. What constitutes a serious adverse drug reaction to the best of your understanding?
7. A reaction that will lead to hospitalization
8. A reaction that resolves on its own
9. A reaction that is life-threatening
10. A reaction that requires another drug treatment
11. Have you heard of pharmacovigilance?
12. Yes ( )
13. No ( )
14. If yes, how did you hear about it
15. Radio ( )
16. Television ( )
17. Newspaper ( )
18. Social media platforms ( )
19. Health care providers (Doctors, pharmacists or nurses)
20. Friends/Relatives
21. Have you ever heard of Adverse drug reactions reporting form (Yellow form)?
22. Yes ( )
23. No ( )
24. If Yes, how did you hear about it?
25. Radio ( )
26. Television ( )
27. Newspaper ( )
28. Social media platforms ( )
29. Health care providers (Doctors, pharmacists or nurses)
30. Friends/Relatives

**SECTION C: ASSESSMENT OF ADVERSE DRUG REACTION REPORTING PRACTICES AMONG PATIENTS**

1. Have you ever experienced an adverse drug reaction?

a). Yes

b). No

2. Action taken in the case of adverse effect/reaction (you may circle more than one option)

a). Informed a healthcare professional

b). Stopped the drug(s)

c). Did nothing because the reaction was tolerable

d). Did nothing because the reaction resolved on its own

e). Used another drug to treat symptoms of reaction

f). Switched to herbal/traditional medicines

g). Switched to another drug

3. Preferred methods of adverse drug reaction reporting (you may circle more than one option)

a). Reporting directly to healthcare professional

b). Phone call or text message

c). Online application designed for adverse drug reaction reporting

d). Filling a reporting form

e). Filling an online reporting form

4. Suggested reasons why patients do not report experienced adverse drug reactions (you may circle more than one option)

a). Do not know the importance of reporting adverse drug reactions

b). Adverse reaction may not be very serious

c). Do not know how to report such reactions

d). Not sure if adverse reaction is related to the medication(s) used

e). Adverse effect/reactions resolved on its own
